# Promoter addresses: revelations from oligonucleotide profiling applied to the *Escherichia coli *genome

**DOI:** 10.1186/1742-4682-2-20

**Published:** 2005-05-31

**Authors:** Karthikeyan Sivaraman, Aswin Sai Narain Seshasayee, Krishnakumar Swaminathan, Geetha Muthukumaran, Gautam Pennathur

**Affiliations:** 1 Centre for Biotechnology, Anna University, Chennai, India; 2 AU-KBC for Research, MIT Campus, Anna University, Chennai, India

## Abstract

**Background:**

Transcription is the first step in cellular information processing. It is regulated by *cis*-acting elements such as promoters and operators in the DNA, and *trans*-acting elements such as transcription factors and sigma factors. Identification of *cis*-acting regulatory elements on a genomic scale requires computational analysis.

**Results:**

We have used oligonucleotide profiling to predict regulatory regions in a bacterial genome. The method has been applied to the *Escherichia coli *K12 genome and the results analyzed. The information content of the putative regulatory oligonucleotides so predicted is validated through intra-genomic analyses, correlations with experimental data and inter-genome comparisons. Based on the results we have proposed a model for the bacterial promoter. The results show that the method is capable of identifying, in the *E.coli *genome, *cis*-acting elements such as TATAAT (sigma70 binding site), CCCTAT (1 base relative of sigma32 binding site), CTATNN (LexA binding site), AGGA-containing hexanucleotides (Shine Dalgarno consensus) and CTAG-containing hexanucleotides (core binding sites for Trp and Met repressors).

**Conclusion:**

The method adopted is simple yet effective in predicting upstream regulatory elements in bacteria. It does not need any prior experimental data except the sequence itself. This method should be applicable to most known genomes. Profiling, as applied to the *E.coli *genome, picks up known *cis*-acting and regulatory elements. Based on the profile results, we propose a model for the bacterial promoter that is extensible even to eukaryotes. The model is that the core promoter lies within a plateau of bent AT-rich DNA. This bent DNA acts as a homing segment for the sigma factor to recognize the promoter. The model thus suggests an important role for local landscapes in prokaryotic and eukaryotic gene regulation.

## Introduction

Transcription, the first step of information flow from DNA, is regulated by sequence specific DNA-protein interactions. The regulation depends on the presence of *cis*-acting elements. The best examples of *cis*-acting elements are promoters. Other well-known examples in bacteria include the Shine Dalgarno (SD) sequence, sigma 32 binding site, LexA binding site, etc.

In bacteria, promoters recognized by sigma factors initiate transcription. The responses of an organism to various stimuli are mediated by changes in gene expression patterns. These changes are initiated by promoter-sigma factor interactions and regulated by other *cis*-acting elements. Thus, families of co-regulated genes are under the control of the same promoter. Though core promoters are small words (6–8 bases), certain changes that are permissible in promoter sequences have little or no effect on their activity. This means that a few closely-related sequences, in the right context, can function as promoters. Identifying promoters is a challenging yet rewarding problem; challenging because promoters can differ subtly in sequence and still retain function, and rewarding because it can shed light on an organism's life style. Computational approaches are required since experimental methods for identifying promoters are not applicable on a genome-wide scale.

In most instances, computational identification or prediction of promoters involves model-based searches. The model is, by and large, derived from prior data. Techniques using artificial neural networks [1] or genetic programming methodologies [2] are also used, and require prior experimental data. Using prior data for identifying new candidates is also known as dictionary-based searching. Databases of experimentally verified *cis*-acting elements are available for promoter prediction [3] through dictionary-based approaches. These approaches are biased towards the best-characterized promoter in the initial dataset, though non-redundant data sets have been used recently [4]. A paucity of experimental data can compromise the efficiency of these methods. The success of dictionary-based methods is directly dependent on the relatedness of the database to the query. It has also been observed that, while using dictionary-based methods, taking into account the local genomic landscape for generating Markov profiles improved the prediction quality in eukaryotes [5]. Another method that has been applied to both simpler and larger genomes is the comparative genome analysis method. It is observed that functional regions, albeit non-coding, are conserved across species and genera. Analyses of this kind have been used for yeast [6,7], higher eukaryotes [8,7] and bacterial regulons [9,7].

In *Saccharomyces cerevisiae*, the distribution of certain words across the genome is non-random. For example, some words appear to be preferred in regions upstream [10] or downstream [11] of genes. Analyses showed that such words occurring preferentially near the genes represent functional elements. Though non-random usage of k-sized words in bacterial genomes has been documented [12,13] in genomic contigs, studies have not focused on the upstream regions of prokaryotic genes.

We have developed a method that uses preferential occurrence of k-sized words within specific (gene-proximal) regions in a given genome to predict *cis-*acting elements. This method does not use a dictionary or database for initiating searches. The method can be applied to any genome of which the gene co-ordinates are known. Its advantage is that there is no extrapolation of data. This allows unique families of *cis*-acting elements for a given genome to be determined. Inter-genome comparison can establish the functionality of conserved words across genera.

The results of oligonucleotide profiling as applied to the genome of *E.coli *K12 [14] are presented. Comparative analyses of the resultant oligonucleotide profiles show that a subset of preferred hexanucleotides in *E.coli*-K12 is conserved across two other genomes, those of *Salmonella typhi *and *Yersinia pestis *[15,16]. We suggest a function for the ubiquitous hexanucleotides that are preferentially present in -100 regions and are neither single-base relatives of TATAAT or AGGA nor CTAG-containing, and we propose a novel model for bacterial promoters.

## Results and Discussion

The results of oligonucleotide profiling, as performed for *E.coli *K12 genome, are discussed. The word size was restricted to six. For higher word sizes the word occurrence frequency was low. Smaller words were not used since the intra-word Markov dependencies, if any, are statistically invalid [17].

**Figure 1:**
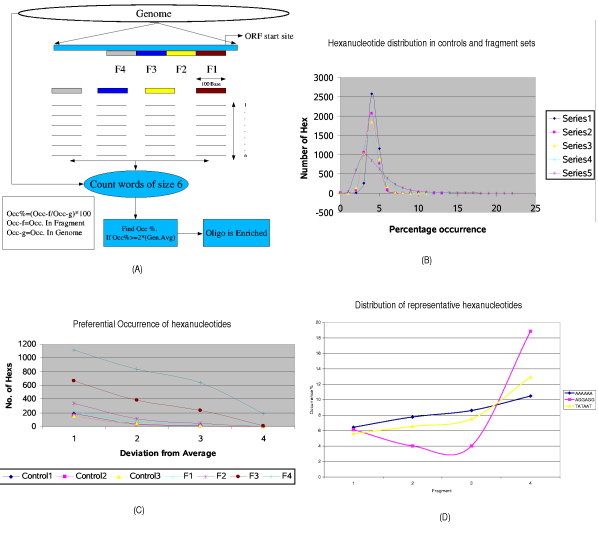
(A) A schematic representation of the procedure used for profiling, incorporating the definition of the four fragments F1, F2, F3 and F4 used in this study. (B) Comparison of the occurrence distribution in the random control (series 1), F4 (series 2), F3 (series 3), F2 (series 2) and F1 (series 4). (C) Number of words whose occurrence is greater than μ+Nσ, where N is on the x axis. (D) Distribution of the three classes of oligonucleotides in the four fragments: TATAAT for class 1, AGGAGG for class 2 and AAAAAA for class 3.

Word occurrences were analyzed in four contiguous sequence sets, F4 through F1 (Fig. 1a),. A threshold of 200% (two-fold increase in occurrence over the genomic average) was set to identify signals for *cis*-acting elements. The average occurrence of a random hexanucleotide in a sequence set is 4.6% of its genomic total and the standard deviation is 0.573. It can be seen that a two-fold increase (9.2%) is more than six times the standard deviation (σ) above the average. Any hexanucleotide that had at least 9.2% of its overall genomic occurrence within any of the four fragments analyzed was termed "enriched" in that respective region. Such enrichment was more pronounced in the gene-proximal regions (-1 to -100 region) than in the distal regions (-300 to -400). In the three random sequence sets (controls), only once did we find enrichment (a CTAG-containing element). Fig. 1a schematically illustrates this procedure.

The preferential occurrences of hexanucleotides within the controls and the fragments under study are contrasted in Table 1. The distributions of hexanucleotide occurrence in control 1 and fragments (F1-F4) are shown in Fig. 1b, while Fig. 1c shows the number of hexanucleotides with frequencies N × (σ) more than average. The units on the X-axis are N (N times σ) and 200%.

**Table 1:** 

Threshold(C)/Region(R)	%>μ+2	%>μ+3	%>μ+4	%>200
	σ	σ	σ	%
Control1	184	42	18	1
Control2	155	30	9	0
Control3	164	46	16	0
F1	229	66	24	7
F2	341	116	44	0
F3	662	387	236	14
F4	1112	834	634	190

The method retrieved 183 hexanucleotides that were enriched in the -100 region. These included the Pribnow box (TATAAT), SD consensus (AGGA), the LexA binding site (CTATNN), sigma 32 binding site one-base relative (CCCTAT) and CTAG-containing regulatory elements [Supplementary Information 1].

The CTAG-containing elements are known to be core repressor binding regions in the Trp, Met and MalPQ operons and the treA gene [18-20]. They occur at high frequency near the rRNA gene clusters [12]. However, in the rest of the genome, we find their distribution to be roughly uniform (data not shown).

Certain trends are apparent in the usage of enriched oligonucleotides by bacterial genomes. The occurrence of some oligonucleotides increases gradually with proximity to genes (class I oligonucleotides), while others (class II oligonucleotides) peak near the genes. A third class comprises non-specifically preferred oligonucleotides (Class III oligonucleotides).

### Class I Oligonucleotide

Bacteria are expected to have limited number of promoter elements and to have them near genes. The Pribnow box in *E.coli *is a representative promoter. The overall frequency of the Pribnow box is lower than the genomic average (1067 occurrences as against the genomic average of ~1400). Here, we analyze: the occurrence of the Pribnow box and its single base substitution relatives, the distribution of the Pribnow box within the -100 region, and the position-dependency of other bases on the Pribnow box in its vicinity. For this analysis, Pribnow box occurrences in the -100 region alone were taken into account for four strains of *E.coli*.

#### Occurrence of Pribnow box

This analysis shows that the occurrence of the Pribnow box increases gradually as one goes closer to genes. Furthermore, seven of its one-base substitution relatives figure in the enriched list [Table 2]. Most of these one-base relatives show a gradual but definite increase in their occurrence as we move nearer the genes [Table 2]. This gives an idea as to how an element that has a function similar to the Pribnow box would behave in other genomes.

**Table 2:** Occurrence of single base relatives of TATAAT in *E.coli *genome. F1:-301 to -400; F2:-201 to -300; F3: -101 to -200; F4: -1 to -100. Those elements that are enriched (> = 200%) are marked by an asterisk in the last column.

Hex	Total Occ.	F4	F3	F2	F1	Occ. % in F1	ENR
TCTAAT	595	29	30	47	46	7.731092	
TAGAAT	507	25	23	36	71	14.00394	*
TATAGT	681	19	38	30	87	12.77533	*
TATAAG	879	45	32	67	102	11.6041	*
TACAAT	903	42	56	71	109	12.07087	*
TATAAC	1590	65	80	94	121	7.610063	
TATGAT	1539	63	111	94	127	8.252112	
TATACT	620	39	40	62	131	21.12903	*
CATAAT	2448	90	106	115	132	5.392157	
TATAAT	1036	58	68	78	134	12.93436	*
TGTAAT	1870	74	80	129	140	7.486631	
TATCAT	2082	101	96	128	164	7.877041	
TATATT	1943	87	130	145	168	8.646423	
TATTAT	2280	118	124	155	178	7.807018	
GATAAT	3735	125	140	172	201	5.381526	
TATAAA	2304	98	144	177	236	10.24306	*
TTTAAT	3671	182	205	219	249	6.782893	
TAAAAT	2947	142	159	204	268	9.093994	
AATAAT	4132	188	201	287	287	6.945789	

#### Distribution of Pribnow box

**Figure 2:**
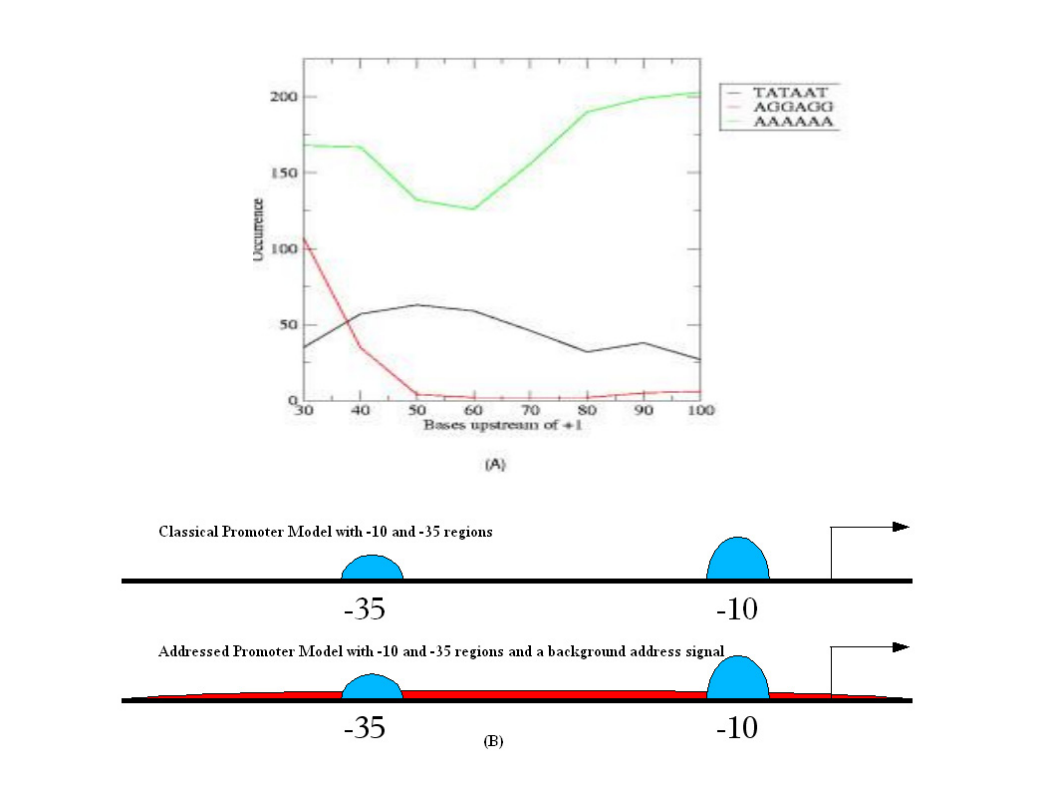
Addressed promoter model. (A) Occurrence distribution of TATAAT, AGGAGG and AAAAAA within the -100 region using a 30-base window: -1 to -30, -10 to -40, -20 to -50, ..., -70 to -100. (B) A schematic comparison of the classical and the addressed promoter models. Blue peaks represent the canonical promoter. Red background (where present) represents the address.

Analyses show that the maximal number of strong minimal promoters occur within the -100 region and that the Pribnow box prefers the -30 to -70 position, centering around -40 [Fig. 2a]. The report by Collado-Vides *et al*. shows that ~80% of the 800 genes analyzed have their promoters in the -100 region. In fact, the highest concentration of promoters that they report is at the -40 region [21], which we corroborate.

#### Markov dependency analysis of sequences surrounding Pribnow box

Markovian analysis of TATAAT-containing sequences (within the -100 region) was done for *E.coli*. For analysis, such sequences were taken from all four *E.coli *genomes (K12, O157:H7, EDL933 and CFT073) to improve statistical significance (TATAAT occurred only 128 times in the -100 region of the K12 genome). The results showed that TTGACA is preferred between positions -32 and -27. Further, it was seen that, with G at -14, the occurrence of TTGACA decreased, (All corresponding data points are highlighted in the Supplementary Information 2 file.) This has been reported by analysis of experimentally characterized promoters [22]. These correlations validate the results of oligonucleotide profiling with respect to the sigma 70 binding site.

### Class II Oligonucleotides

AGGA- (SD consensus) and CTAG-containing hexanucleotides belong to this class. Unlike the Class I oligonucleotides, Class II oligonucleotides show a steep increase in occurrence in the -100 region. This is expected in the case of the Shine-Dalgarno sequence (AGGA), since it should lie within 30 base pairs upstream of the ORF start site (owing to geometric constraints imposed by the ribosomal complex).

Another example of this class is the tetranucleotide CTAG, representing all the hexanucleotides that contain it. CTAG kinks DNA when bound by proteins [23], making it a likely candidate for a regulatory site. CTAG also has low genomic frequency, uniform distribution and a preference for the -100 region. This might imply a global regulatory function.

### Class III Oligonucleotides

Certain oligonucleotides not only have a more than average genomic frequency but are also more common in the -100 region. Many of these are A/T rich oligonucleotides, which are known to bend DNA when present in stretches [24]. The presence of such A/T repeat elements upstream [25] and downstream [26] of the canonical promoter is necessary. They are evidently not stand-alone signals. We propose that they are facilitator elements that are necessary but not sufficient for promoter recognition and function. The set of such oligonucleotides that were readily distinguished as facilitators is given, along with their distribution, in Supplementary Information 3. They occur preferentially up to -100 and beyond. We find this significant since a recent report shows that DNA of size 90 base pairs can bend upon itself in a sequence-dependent manner [24].

Though all 64 A/T containing hexanucleotides were found to occur more frequently than the genomic average, only 18 of them were enriched in the -100 region. Thus, the increased occurrence of Class III hexanucleotides is not an artifact of increased base frequency. It transpires that the genome increases the bending capacity of the -100 region by preferential usage of certain oligonucleotides.

The occurrence of hexanucleotides representing each of the three classes is shown in Fig. 1d. TATAAT is used to represent class I, AGGA-containing hexanucleotides to represent class II and AAAAAA to represent class III.

### Protein Binding Capacity of the -100 region: Evidence from NDB

We analyzed the occurrence of enriched hexanucleotides in a protein-bound state in the NDB database [27]. Of the ~130 hexanucleotides that are neither TATAAT-related (1 base substitution oligonucleotides) nor AGGA- or CTAG-containing, 112 have at least one occurrence in the database, bound to proteins [data not shown]. Most of them occurred more than once in the database in a protein-bound state. These results show the propensity of the genome to increase the protein-interacting capacity of the -100 region and hence increase the activity of this region.

### Dependency Analysis

A position-specific probability matrix (PSPM) was created for enriched oligonucleotides that were not TATAAT related or AGGA/CTAG-containing. This matrix was used to determine the tendency of hexanucleotides to assume specific consensus words within the -100 region of the genes. Secondary matrices were derived by anchoring the first base in the PSPM. The consensus words derived from these matrices are given in Supplementary Information 4. For each secondary matrix, two more character states were chosen for anchoring on the basis of their prominence,. The results show a strong preference for tetra-A signals, TATA-containing signals and GGA-containing signals.

### Inter-genome comparison of hexanucleotide usage profiles

Conservation of DNA sequence across genomes has been established as a pointer to functionality. This method has been used to identify regulatory regions in *Saccharomyces *[6] by sequence comparison among different species. We see that the logic extends beyond conservation of sequences and patterns to that of oligonucleotide profiles.

We have compared the profile of enriched hexanucleotides between *E.coli*, *Salmonella enterica *and *Yersinia pestis *to test its validity. The *E.coli *and *Salmonella *profiles shared 110 enriched oligonucleotides out of 160 in *Salmonella typhi*. *Yersinia pestis*, whose profile had 97 enriched oligonucleotides, shared 66 of them with *E.coli*. Of those that were conserved across genomes, the AGGA-containing and CTAG-containing hexanucleotides, TATAAT, and the LexA binding site were prominent (Supplementary Information 5).

While conservation of hexanucleotides usage implies functionality, the converse may not be true and might reflect unique regulatory / facilitator elements for each genome.

### Role of facilitator elements in promoter identification and the Addressed Promoter Model

Classical promoters in bacteria are sigma factor binding sites. The sequence that is known to bind to sigma factor with maximal affinity in vitro is taken to be the strongest promoter. DNA footprinting experiments do not allow us to assess the importance of the surrounding sequences.

It is clear from the profiles that the strongest promoters have limited occurrence in the genome. Most genes are controlled by sigma 70 in *E.coli *[28], and only ~12% of the overall strong consensus occur in a region where they are maximally effective [21]. The question to be addressed is how a sigma factor (Sigma 70 in this case) can distinguish the promoter from non-specific promoter-like signals (degenerate -10 and -35 like signals in non-functional places in the genome). The sigma factor could not read every one of the possible signal combinations since this would result in enormous loss of time in bacterial genomes. In larger genomes, given the small size and degeneracy of the promoters, it is possible that the sigma factor would recognize a false signal on most occasions.

To account for the efficiency of promoter recognition in the organism, we propose the addressed promoter model, where the sigma factor binding element is an information-dense peak (specific information) within a plateau of moderate information density (different but related words). The peak and the plateau together constitute the promoter. The plateau is formed by class III oligonucleotides that have the capacity to bend DNA. The facilitators are an integral part of the promoter. The presence of facilitators, which occur in greater frequencies around the core promoter, will serve as addresses for the core promoter. These addresses act as homing segments that allow the transcription factor to recognize the core promoter and bind to it.

This model immediately suggests a way of identifying *cis*-acting regions in eukaryotes, where greater genome sizes and more degeneracy are seen. The extension of this logic would be to view enhancers and other regulatory regions in large eukaryotic genomes as local landscapes rather than as sequence motifs. While the protein binding sites would still be sequence motifs, their occurrence in a particular landscape may prove to be the determining factor for their activity. This accords with the observation of Huang et. al. [5] that local genomic landscape information affects the prediction quality of promoter elements.

To illustrate this model, we have analyzed the distribution of one representative element from each of the three classes. The distribution was studied in a 30-base sliding window with a 10-base pitch. The representative elements are TATAAT (Class I), AGGAGG (Class II) and AAAAAA (Class III). The distribution is shown in Fig. 2a. It can be seen that AAAAAA forms a plateau around the TATAAT peak. The classical model and the addressed promoter model are contrasted in Fig. 2b.

## Conclusion

This method for identifying regulatory regions in DNA is powerful. Its strength is its ability to use the genomic sequence as a control. This obviates the need for data extrapolation from related genomes. The method can identify functional elements that can be experimentally characterized.

Application of this method to the *E.coli *K12 genome reveals the presence of at least three classes of *cis*-acting elements. The occurrence, distribution and dependencies of these elements have been analyzed. Most of the profile data correlate with existing experimental evidence. The canonical sigma70 promoter has been analyzed in further detail in four *E.coli *genomes.

The information derived from *E.coli *K12 using this method suggests that the functionality of a promoter is determined not only by the sequence of the core promoter element but also by its local milieu. We note that the occurrence of proposed facilitator elements extends just beyond the length known for DNA to bend upon itself (90 bp) and this, together with other reports about AT-rich tracts in the vicinity of the canonical promoter, suggests that the sigma factor recognizes a promoter more efficiently if it is present in the "address" region. This immediately explains why the transcription process is efficient in spite of the degeneracy that the promoter exhibits. We see that the occurrence of facilitators is not an artifact of increased base frequencies.

The occurrence of many of the enriched hexanucleotides as protein-bound DNA complexes in the NDB database is indicative of their protein-interacting ability. This reflects on the protein binding capacity of the gene proximal regions in *E.coli *K12.

The limitation of this method is its inability to pick up rare regulatory elements. In small genomes the method is known to give false positives, and in degraded genomes it picks up false negatives. In such cases, comparative analysis with related genomes will give valuable information.

## Methods

### Sequence Extraction

Published genome sequences from the NCBI database (.fna file) were used. The start sites of genes given in the annotation file (.ptt file) were used for extracting upstream sequences of all the genes. Upstream sequences were taken only from their respective strands (+ strand for + genes and vice versa) because of the directionality of promoters. Four such fragments were taken from upstream of each gene, viz. -1 to -100, -101 to -200, -201 to -300 and -301 to -400. The distance between any two genes was not given importance because of the possibility that regulatory and transcriptional start sites may be present in the coding region of the preceding gene.

### Profiling

For every gene in the *E. coli *K12 genome, four contiguous DNA fragments from the corresponding strand were extracted. The length of each fragment was 100 bases. The fragments were named F4 through F1, where F1 is the gene-proximal fragment. There are 4311 genes in *E.coli*. Four sequence sets, one each for F1, F2, F3 and F4, were created for all the genes. Each of these sequence sets covers approximately 4.6% of the genome.

Occurrence of all hexanucleotides was counted on both strands of the genome and the four upstream-sequence sets. The Compseq program from the EMBOSS [29] suite was used for this purpose. Any word that was non-functional was expected to be distributed equally across the sequence sets. Thus, for a non-functional word in the upstream context, we expected approximately 4.6% of its genomic occurrence in any of the sequence sets.

Since *cis*-acting elements are gene-proximal, we expected their occurrence to be higher in F1 than elsewhere. We set a threshold (T) of 200% in word frequency to identify signals. Given a standard deviation of 0.56, it is apparent that a 200% increase (9.2% of genomic occurrence) is more than 6σ, which is significant. Words whose frequency in a given sequence set was 9.2% or more were termed "enriched" in the corresponding fragment.

All analyses were carried out using Perl 5.6.1 scripts on a Mandrake Linux 9.1 platform. The complete dataset is available in an in-house MySql -based server.

### Markov Dependency Analysis

We analyzed the character-state probabilities of all the words (137 words) for which a function could not be assigned. For this, we created a position-specific probability matrix (PSPM). The PSPM was derived from a position-specific frequency matrix (PSFM), which is defined as follows. For a word size of L, a PSFM is a 4 × L matrix M, where each element M_i,j _[i ∈ {A,T,G,C} and j ∈ {1,2, ... L}] is the number of times the character state i occurs at position j. In this case, L = 6.

If S is the sum of all occurrences of words, then the PSPM is related to the PSFM as given below:

PSPM = (1/S) × PSFM

Such a matrix was used to derive consensus words preferred in the -100 region. From the PSPM, four sub-matrices were derived by anchoring the various character states (A, C, G, and T) at the first position. Further dependencies were analyzed by subsequent anchoring of two more positions, based on their prominence in the sub-PSPMs, to their representative character states.

### Markov Analysis for TATAAT-dependent Signals

For each occurrence of the Pribnow box within the -100 region, the preceding 50-base region was extracted. The PSPM was created for the sequence set as described above, where the value of L is 50. Different profiles were created by anchoring the base profile at all positions with all four bases. This was used to analyze the dependency of upstream signals on TATAAT. This analysis was done on a sequence set collated from all the four strains of *E.coli*.

## Authors' contributions

KS gave the core idea for oligonucleotide profiling, analysis of occurrence and proposed the model. ASNS worked with KS in profiling and analyzing the statistical significance of results, and KrS worked with KS in analyzing the distribution of words in gene proximal regions. GM was involved in analysis of results and critically analyzing the manuscript. PG is the group leader.
